# Role of an Implementation Economics Analysis in Providing the Evidence Base for Increasing Colorectal Cancer Screening

**DOI:** 10.5888/pcd17.190407

**Published:** 2020-06-25

**Authors:** Sujha Subramanian, Florence K.L. Tangka, Sonja Hoover

**Affiliations:** 1 RTI International, Waltham, Massachusetts; 2 Division of Cancer Prevention and Control, Centers for Disease Control and Prevention, Atlanta, Georgia

## Abstract

**Purpose and Objectives:**

Since 2005 the Centers for Disease Control and Prevention (CDC) has funded organizations across the United States to promote screening for colorectal cancer (CRC) to detect early CRC or precancerous polyps that can be treated to avoid disease progression and death. The objective of this study was to describe how findings from economic evaluation approaches of a subset of these awardees and their implementation sites (n = 9) can drive decision making and improve program implementation and diffusion.

**Intervention Approach:**

We described the framework for the implementation economics evaluation used since 2016 for the Colorectal Cancer Control Program (CRCCP) Learning Collaborative.

**Evaluation Methods:**

We compared CRC interventions implemented across health systems, changes in screening uptake, and the incremental cost per person of implementing an intervention. We also analyzed data on how implementation costs changed over time for a CRC program that conducted interventions in a series of rounds.

**Results:**

Implementation of the interventions, which included provider and patient reminders, provider assessment and feedback, and incentives, resulted in increases in screening uptake ranging from 4.9 to 26.7 percentage points. Across the health systems, the incremental cost per person screened ranged from $18.76 to $144.55. One awardee’s costs decreased because of a reduction in intervention development and start-up costs.

**Implications for Public Health:**

Health systems, CRCCP awardees, and CDC can use these findings for quality improvement activities, incorporation of information into trainings and support activities, and future program design.

**Summary**

**What is already known on this topic?**

The Community Preventive Services Task Force recommends implementation of several single and multilevel interventions to improve colorectal cancer screening uptake.

**What is added by this report?**

We compared single and multilevel colorectal cancer interventions implemented across health systems, improvements in screening uptake, and the incremental cost per person of intervention implementation. We also looked at how implementing rounds of interventions can change costs.

**What are the implications for public health practice?**

The Colorectal Cancer Control Program’s Learning Collaborative generated evidence to guide the implementation of colorectal cancer interventions in health systems, and in particular health systems that serve a large proportion of underserved populations.

## Introduction

The rate of colorectal cancer (CRC) screening has been increasing nationally, and by 2016, 67.7% of the US population was up to date with CRC screening recommendations (1,2). However, the prevalence of CRC screening is lower than the 2016 national average among some populations, such as racial and ethnic minority groups, people who have no health insurance or who are underinsured, and people residing in some geographic areas (3–5).

The Centers for Disease Control and Prevention (CDC) has implemented several initiatives to increase uptake of CRC screening. In 2009, CDC began a national program of promoting and providing CRC screening services through its awardees in the Colorectal Cancer Control Program (CRCCP) (https://www.cdc.gov/cancer/crccp/index.htm) (6). Since 2015, the program has focused on increasing CRC screening prevalence in health systems through evidence-based interventions recommended by *The Community Guide* (7). The CRCCP has focused on 4 priority evidence-based interventions: reducing structural barriers, provider assessment and feedback, patient reminders, and provider reminders. These interventions can also be undertaken alongside supporting activities, such as patient navigation, small media (eg, brochures), and professional development and training. In 2015, CRCCP funded 30 awardees: 23 state health departments, 1 tribal organization, and 6 academic medical centers.

## Purpose and Objectives

**Figure 1:**
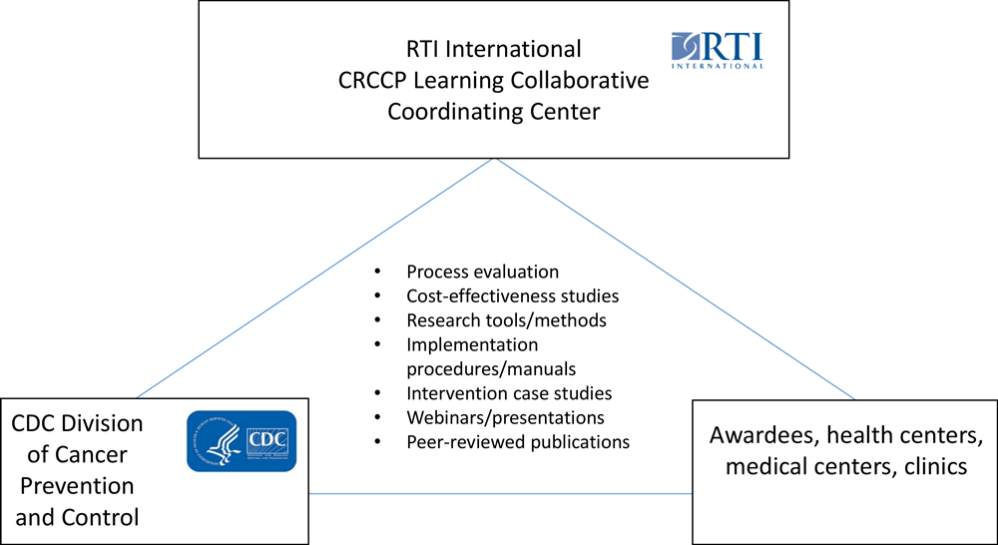
Overview of the Colorectal Cancer Control Program Learning Collaborative. Abbreviations: CDC, Centers for Disease Control and Prevention; CRCCP, Colorectal Cancer Control Program.

In 2016, CDC and RTI International (www.rti.org) began work with a subset of 14 of the CRCCP’s 30 awardees to create the CRCCP Learning Collaborative. The objective of the Learning Collaborative is to work with awardees to analyze implementation, effectiveness, and cost-effectiveness of the evidence-based interventions, supporting activities, and other interventions implemented by the awardees to improve CRC screening uptake (Figure 1) (8,9). As of 2020, 14 awardees agreed to participate in the CRCCP Learning Collaborative: 9 health departments, 4 academic medical centers/universities, and 1 tribal organization (8). CDC sets the goals and oversees the CRCCP Learning Collaborative, and RTI serves as a coordinating center and provides logistical, coordination, and scientific support for collecting data, conducting economic assessments, and disseminating findings.

For this study, we identified a further subset of the 14 awardees: CDC and RTI analyzed data from 7 awardees and their implementation sites (for a total of 9 implementation sites); because they agreed to participate in this study, these 7 awardees and their implementation sites represent a convenience sample. Most health departments and academic medical centers partnered with federally qualified health centers (FQHCs), which serve low-income populations; 1 health department partnered with Medicaid managed care plans and their members. The 8 participating FQHCs are in both urban and rural areas; the size of the population eligible for CRC screening and the number of clinics vary by FQHC (range, 1–9 clinics). The objective of our study was to determine how findings from the economic evaluations of these 7 awardees and their implementation sites can drive decision making, improve program implementation, and facilitate program diffusion to other health care delivery settings. We conducted our analysis in 2019–2020. The studies were implemented by CDC’s CRCCP awardees. RTI institutional review board approval was not required for this evaluation study because it used nonidentifiable data and did not constitute human subjects research.

## Intervention Approach

**Figure 2:**
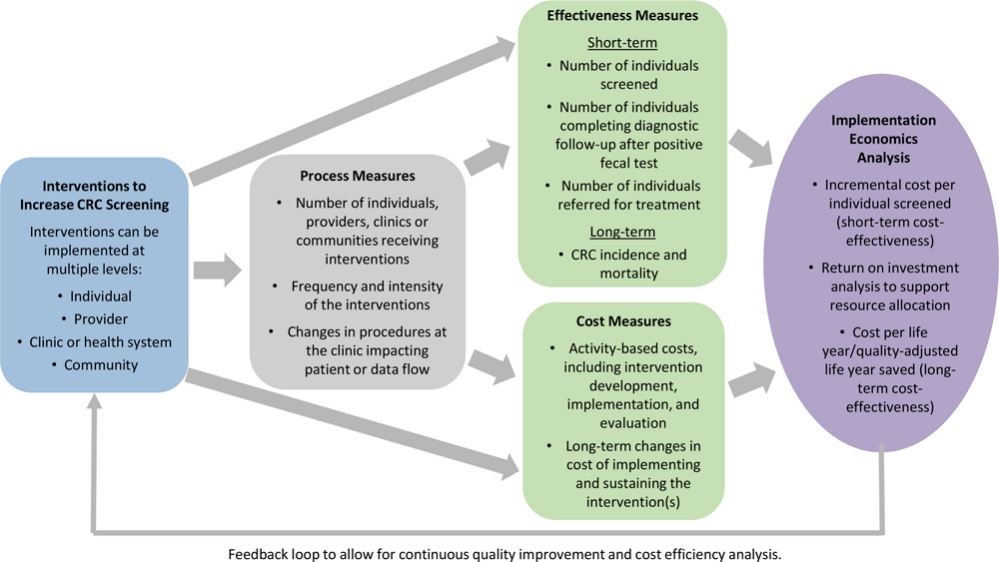
Framework for the implementation economics evaluation used by the Colorectal Cancer Control Program Learning Collaborative. Abbreviation: CRC, colorectal cancer.

The CRCCP Learning Collaborative used an “implementation economics” evaluation framework (Figure 2). We previously defined implementation economics as a “subdiscipline of implementation science that focuses on cost-related economic evaluation (cost-of-illness analysis, program cost analysis), cost-effectiveness, cost–benefit analysis, cost utility, budget impact, and cost minimization” (10). Implementation economics has a broader perspective than static cost-effectiveness analysis and involves systematic assessment of processes, effectiveness, costs, and cost-effectiveness analyses over time. Implementation economics analyses include estimating incremental cost per person screened, determining return on investment, and assessing long-term cost-effectiveness. It considers dynamic changes that occur over time across program implementation phases. Interventions can be implemented at multiple levels, including patient, provider, clinic, and community. Most health systems participating in the CRCCP Learning Collaborative implement multicomponent interventions. A key feature of the implementation economics framework is the collection of process measures both to track the steps involved in implementing interventions and to quantify the intensity (ie, types of evidence-based interventions and frequency implemented) of the intervention components. The 14 CRCCP Learning Collaborative participants collected numerous types of process measures (Table 1).

**Table 1:** Process Measures Collected From Colorectal Cancer Control Program Learning Collaborative Partners^a^

Intervention	Process Measure
One-on-one education	• Number of patients educated
Fecal immunochemical test (FIT) or fecal occult blood test (FOBT) mailings	• Number of FIT/FOBT kits mailed • Number of FIT/FOBT kits returned • Number of FIT/FOBT kits returned after reminders
Provider reminders	• Number of providers targeted • Frequency of reminders by approach (paper-based or electronic notification)
Patient navigation	• Number of reminders made by approach (telephone calls, in-person meetings, emails, or text messages) • Frequency of education provided by approach (telephone or in-person) • Number or proportion of no-shows or missed appointments • Proportion of late cancellations (within 24 or 48 hours of appointment) • Proportion of patients with adequate bowel preparation • Types of services provided to patients to overcome barriers to care
Client or provider incentive	• Number and value of incentives provided • Frequency of incentives
Patient reminders	• Number of reminders done by approach (eg, telephone, letter) • Number of repeat reminders by approach • Number of FIT kits returned after reminder by approach
Provider assessment and feedback	• Number of providers receiving reports • Frequency of providers receiving reports • Time intervals reported in reports

a The objective of the Colorectal Cancer Control Program Learning Collaborative is to work with awardees to analyze implementation, effectiveness, and cost-effectiveness of the evidence-based interventions, supporting activities, and other interventions implemented by the awardees to improve colorectal cancer screening uptake (8,9).

## Evaluation Methods

As of 2019, the effectiveness measures we used included short-term effects, and emphasized number of people screened. The collection and reporting of data for other measures along the continuum of screening, such as the proportion of people completing diagnostic follow-up tests and treatment referrals, was challenging for clinics to collect completely and accurately because these services are often provided outside their facility. The collection of those data is targeted for future process improvement, because it is essential to track effectiveness measures along the entire screening continuum to assess the effectiveness of interventions on patient outcomes. We plan to assess long-term effects by using a validated microsimulation model (11–13). All participants collected detailed activity-based cost information following a standard methodology previously developed for CRC screening evaluations (14,15); however, we tailored the tools for collecting data on costs for awardees to reflect implementation of their interventions. 

We conducted all analyses in real-world settings and used pre–post comparisons. Although the time frame of implementation differed across the health systems, we used standardized approaches to estimate cost per person screened at each health system. We used predefined time periods for the numerator and denominator to derive the effectiveness measures at a given health system, and we used the same time period to generate cost estimates. We collected data on costs during several years or periods to understand changes in intervention costs with sequential implementation of the interventions. Additionally, we collected cost data regardless of funding source because many of the partners have both internal and external sources to supplement CRCCP funding; for example, some of the intervention evaluations involved incentive payments, which are not covered by the CRCCP.

We compared the range of improvements observed in screening uptake in our study with the range of improvements in multicomponent CRC screening interventions reported by the Community Preventive Services Task Force (CPSTF) (16).

All analyses provide feedback to clinic staff members to evaluate processes and identify approaches to improve the effectiveness and cost-effectiveness of their interventions. Because the objective of the cost data collection was to quantify costs associated with the implementation of the evidence-based interventions, we did not collect separate data on the clinical cost of the 2 screening methods, fecal immunochemical test (FIT) and colonoscopy.

## Results

### Comparison of costs across health systems

The CRCCP Learning Collaborative awardees implemented various evidence-based interventions, supporting activities, and other interventions. We summarized results from 6 awardees and implementation sites that participated in implementation economics evaluations (Table 2). Five awardees implemented 1 intervention each; 3 awardees implemented multiple interventions through its FQHCs. Of these 3 awardees, 1 implemented patient reminder interventions in 9 of its FQHCs. For ease of comparison, we included data from only 1 of the 9 FQHCs, as the cost per person screened at this FQHC closely reflected the median cost per person screened among the 9 FQHCs. The FQHCs of the other 2 awardees that partnered with FQHCs implemented multicomponent interventions that consisted of patient reminders, provider assessment reports, and provider feedback reports. One FQHC included provider reminders as part of the multicomponent interventions. Two awardees began incentive programs, one for support staff (eg, office staff, medical assistants, laboratory technicians) and one for patients. Support staff members received monetary incentives of $25 each when screening prevalence met certain screening rate thresholds or increased by 5 percentage points (eg, 55% to 60%). Patients received gift cards ($10 value) upon completion of either an FIT, a fecal occult blood test (FOBT), or a colonoscopy.

**Table 2:** Description of Intervention, Effectiveness, and Cost by Awardee in the Colorectal Cancer Control Program Learning Collaborative^a^

Awardee and Implementation Site	Intervention	Time Frame	No. of Clinics	Increase in FIT Kits Returned or Screens Completed From Baseline^b^	No. of People Eligible for Intervention	Change in Overall FQHC Screening Uptake From Baseline to Implementation Period (Percentage-Point Increase)^c^	No. of People Screened Attributable to Intervention(s)^d^	Intervention Cost, $	Cost per Person Screened, $
1	Mailed FIT (supplemented with outreach activities to increase FIT uptake)	1 y	9	31.0% of FIT kits returned	5,178	52.2% to 59.3% (7.1)	1,607	30,148	18.76
2	Patient reminders	1 y	2	17.2% increase in FIT kits returned	541	15.4% to 42.1% (26.7)	93	6,897	74.16
3	Provider incentives	18 mo	9	27.6% more FIT screens than at baseline	Not available^e^	51.9% to 56.8% (4.9)^e^	1,998	133,447	66.79
4	Provider reminder (supplemented by patient reminder and provider assessment and feedback)	21 mo	9	21.2% increase in screens	1,334	27.8% to 37.4% (9.6)	283	40,909	144.65
5	Multicomponent interventions that include patient reminders and provider assessment and feedback	1 y	1	Not available	1,858^f^	38.3% to 57.2% (18.9)	332	13,278	40.00
6	Multicomponent interventions that include patient and provider reminders and provider assessment and feedback	2 y^g^	9	Not available	8,626^f^	21.1% to 39.2% (18.1)	2,533	60,224	23.78
7	Multicomponent interventions that include patient reminders and provider assessment and feedback	3 y	4	Not available	4,771	25.7% to 35.4% (9.7)	943	27,497	29.16
8	Multicomponent interventions that include patient incentive, patient navigation, and patient reminders	13 mo	1	25.9% increase in FIT kit return rates	353	19.0% to 39.0% (20.0)	91	12,250	134.61

Abbreviations: FIT, fecal immunochemical test; FQHC, federally qualified health center.

a The objective of the Colorectal Cancer Control Program Learning Collaborative is to work with awardees to analyze implementation, effectiveness, and cost-effectiveness of the evidence-based interventions, supporting activities, and other interventions implemented by the awardees to improve colorectal cancer screening uptake (8,9).

b Percentage increases in FIT kit return rates or selected CRC screens among people targeted by selected interventions implemented by the FQHC. Three FQHCs did not have this information because the interventions were not directly tracked, and only overall clinic CRC screening rate numerator and denominator were available to produce an overall CRC screening uptake.

c The denominator for this rate was the entire cohort eligible for CRC screening at the FQHC.

d The number of people screened was based on the percentage increase in screening that was attributable to the intervention in the 5 FQHCs. In the 3 FQHCs that did not have this information, the number of people screened was based on the overall change in the numbers screened in the FQHC (percentage-point increase is shown in Column 7).

e The number of people screened was based on average differences in monthly screens during the baseline and implementation periods. Therefore, the number eligible cannot be directly calculated from the increase in screening rate and number screened.

f Estimated number of eligible patients calculated based on baseline period. Estimate used as numerator and denominator can change because of changes in FQHC patient population.

g Average across implementation years.

We tracked the direct effect of the interventions implemented by the 5 awardees that implemented only 1 intervention each. The direct effectiveness of these interventions on CRC screenings was an increase from 17.2% to 31% . Across all interventions and multicomponent interventions, increases in the overall FQHC screening uptake ranged from 4.9 to 26.7 percentage points. Each FQHC implemented interventions that increased CRC screening among its low-income population. The populations served by the FQHCs varied by race, age, and other factors, such as primary language spoken; therefore, changes in screening uptake by sociodemographic characteristics across FQHCs are not directly comparable.

The multicomponent interventions described by the CPSTF increased CRC screening uptake by a median of 15.4 percentage points when all recommended tests were considered, by 10.2 percentage points for colonoscopy screening, and by 7.7 percentage points for FOBT screening, compared with no intervention (16). The CPSTF reported results for multiple types of studies, including randomized control trials, and, therefore, the findings may not be directly comparable with the findings of our real-world assessment. In our study, FQHCs with a higher prevalence of screening at baseline were generally likely to achieve smaller increases in screening uptake compared with FQHCs with a lower prevalence of screening at baseline. In FQHCs in which more than half of the eligible population was up to date with screening, the increases in screening uptake were about 7 percentage points or less, whereas in FQHCs where less than half the population was up to date, the increases were about 10 percentage points or more.

Across the FQHCs, the cost per person screened ranged from $18.76 to $144.65. These estimates were lower than the estimates reported in the CPSTF review, in which the median incremental cost per person screened was $582.44 (interquartile interval, $91.10–$1,452.12 [2016 $US]) (16). These estimates may not be directly comparable because of the differences in the study implementation periods and design; also, not all FQHCs in our study implemented multicomponent interventions. Additionally, no benchmarks exist for determining the efficient use of resources for CRC screening promotion at the clinic level. Our cost estimates provide FQHCs with information to assess the resources required to implement interventions and fulfill performance reporting requirements mandated by the Health Resources and Services Administration and to increase CRC screening to meet national targets, such as the Healthy People 2020 target of increasing the proportion of adults who receive a colorectal cancer screening based on the most recent guidelines to 70.5% (17) and the National Colorectal Cancer Roundtable’s 80% Pledge (18). Additionally, our analyses offer health systems a data-driven approach to monitor their own performance and to use activity-based costs to identify approaches to improve implementation processes to further increase cost-effectiveness of the interventions.

In general, the cost per person screened at FQHCs participating in our study tended to be lowest in those with large target populations. All interventions have fixed implementation costs; therefore, the greater the number of persons screened because of the intervention, the lower the cost per person screened. The performance of CRC screening interventions cannot be directly compared between clinics with small target populations and clinics with large target populations. For instance, rural clinics that tend to serve a small catchment area will likely have higher per-person costs than urban clinics that tend to serve a large catchment area. Furthermore, differences in the length of the implementation periods is another potential reason for variation in cost per person screened across FQHCs.

### Changes in cost across implementation periods

A CRC program that conducted interventions in a series of rounds (19) illustrates how program implementation costs may decrease over time. In that program, the awardee implemented a mailed reminder to increase CRC screening uptake among the Medicaid Managed Care population in 2 regions of the state. The reminder was mailed to 2 groups. One group received the reminder with an incentive offer; the second group received the reminder with no incentive offer. After completion of CRC screening (either an FOBT, sigmoidoscopy, or colonoscopy), patients in the reminder-plus-incentive group were given a $25 gift card. The reminder-plus-incentive program was implemented in 2 rounds. In each round, Region 1 was first and Region 2 was second. The program was implemented from February 2016 through November 2016 and February 2017 through January 2018 in Region 1, and from September 2016 through February 2017 and October 2017 through July 2018 in Region 2.

In Region 1, costs were less in each cost category in Round 2, except for incentive payments (Table 3). Incentive payments are directly related to target population size and response rate; therefore, we expected the cost of incentives to vary. Overall, costs in Region 1 were $121,000 (68.7%) less in Round 2 than in Round 1, despite a longer implementation period in Round 2. Decreases in cost ranged from $3,712 (36.6%) for patient recruitment and tracking to $70,044 (81.2%) for administration and management. Region 2 followed a similar pattern of general decreases in costs between rounds, except for a small increase of $524 (6.5%) for patient recruitment and tracking. Overall costs were less in Region 2 than in Region 1 in each round, likely because of efficiencies gained from Region 2 following Region 1 in implementation and overlapping interventions during Round 2. Intervention development, administration, and evaluation are likely to have substantial fixed start-up costs; therefore, we were not surprised to see these costs decrease considerably between rounds of implementation. These findings highlight the importance of tracking costs over multiple iterations of intervention implementation. Costs not directly related to volume of patients served are likely to decrease as information learned is incorporated into implementation activities.

**Table 3:** Difference in Costs of Intervention Activities Between Round 1 and Round 2 of 1 Participant in the Colorectal Cancer Control Program Learning Collaborative^a^

Category	Region 1^b^				Region 2^c^			
	Round 1 (February 2016–November 2016)	Round 2 (February 2017–January 2018)	Difference Between Round 1 and Round 2	Change, %	Round 1 (September 2016–February 2017)	Round 2 (October 2017–July 2018)	Difference Between Round 1 and Round 2	Change, %
**Number of mailings**	7,123	6,981	142	2.0	10,943	9,802	1,141	10.4
**Number of months**	9	11	−2	−22.2	5	9	−4	−80.0
**Costs, $**								
Intervention development^b^	52,154	11,664	40,490	77.6	28,215	9,278	18,937	67.1
Patient recruitment and tracking	10,150	6,438	3,712	36.6	8,112	8,636	−524	−6.5
Incentive payment	5,603	6,352	−749	−13.4	6,004	5,993	11	0.2
Administration and management	86,224	16,180	70,044	81.2	28,206	19,158	9,048	32.1
Evaluation and reporting	21,874	14,371	7,503	34.3	11,983	5,904	6,079	50.7
Total cost	176,005	55,005	121,000	68.7	82,520	48,968	33,552	40.7

a The objective of the Colorectal Cancer Control Program Learning Collaborative is to work with awardees to analyze implementation, effectiveness, and cost-effectiveness of the evidence-based interventions, supporting activities, and other interventions implemented by the awardees to improve colorectal cancer screening uptake (8,9).

b The implementation period began with the mailing of the letters, continued as screenings were tracked, and ended when the evaluation period was completed.

c The intervention development period in Region 1 was from February 2015 through January 2016 (12 months) in Round 1 and November 2016 through February 2017 (4 months) in Round 2. The intervention development period in Region 2 was from March through September 2016 (7 months) in Round 1 and August through October 2017 (3 months) in Round 2.

## Implications for Public Health

The CRCCP Learning Collaborative findings were disseminated through various approaches, including webinars, presentations, peer-reviewed articles, 1-pagers, and training sessions. Many of these dissemination approaches offer interactive forums for discussion of the implications of results and the generalizability of the findings to various types of settings. The findings from the implementation economics evaluations offer lessons for improving CRC intervention implementation at multiple levels. First, the clinics and health systems used the data generated through participation in the CRCCP Learning Collaborative to initiate continuous quality-improvement processes. Activity-based cost data provided quantitative information for pursuing data-driven strategies to improve efficiency in implementing interventions to increase CRC screening. Second, the clinics provided feedback on lessons learned to the CRCCP awardees (generally state health departments and academic partners), who then incorporated this information into their trainings and support activities with other health systems. The CRCCP awardees were liaisons in translating and transmitting findings among the health systems they interact with over multiple years of the programs. Third, at the CDC level, the findings from this economic evaluation can be used to design future programs. For example, analysis of the previous round of the CRCCP showed that a large proportion of funds was used for mass media promotion, an intervention not recommended by the CPSTF because of insufficient evidence of effectiveness (10). The results partially informed CDC’s design of the second round of CRCCP, where awardees select and implement multiple evidence-based interventions. Fourth, decision makers and legislators at the federal and state levels can use findings from the CRCCP Learning Collaborative as evidence for evaluating the effect of CRC interventions and reaching informed funding decisions.

We acknowledge several limitations to this research. First, the CRCCP Learning Collaborative is a convenience sample, a subset of all awardees of the CRCCP. Because of this, findings may not be representative of all CRCCP awardees. Results should not be compared across the Learning Collaborative because of differences in FQHCs in terms of populations, sizes, and geographic locations. Second, we used data on the number of patients screened as the primary short-term measure of effectiveness. Other measures of effectiveness exist along the continuum of screening services, but we did not use them because many FQHCs do not provide these services and thus would be unable to collect these data. We also reported results for FQHCs that had a short implementation time frame, and we recognize that long-term effectiveness may change as the intervention continues. Lastly, much of the cost data were collected retrospectively by FQHC staff members, and these data may be subject to recall bias.

Despite these limitations, the CRCCP Learning Collaborative has generated evidence to guide the implementation of CRC interventions in health systems that serve a large proportion of low-income people who are predominantly from racial/ethnic minority groups. Our study supports the findings of the American Cancer Society’s Community Health Advocates Implementing Nationwide Grants for Empowerment and Equity (CHANGE) grant program, which funded FQHCs to promote CRC screening (20). In that study, funded FQHCs that implemented evidence-based interventions increased CRC screening uptake at a higher rate than nonfunded FQHCs that may have implemented interventions. Increasing CRC screening among FQHC patients can substantially reduce racial/ethnic disparities and improve CRC outcomes. Although strides in reducing racial/ethnic disparities have been made, we plan to address challenges related to improving data quality and collecting comprehensive data along the screening continuum in future implementation economics evaluations. A key strength of the CRCCP Learning Collaborative is the opportunity to build on past evaluations to inform future program designs and interventions and the creation of a continuous learning environment to improve the implementation of interventions to increase uptake of CRC screening.
